# Maternal Multiple Sclerosis and Offspring’s Developmental and Behavioral Profile: A Case-control Study

**DOI:** 10.2174/011570159X370866250416103923

**Published:** 2025-07-04

**Authors:** Martina Siracusano, Doriana Landi, Elisa Carloni, Chiara Scoppola, Marialaura Ferrara, Assia Riccioni, Lucrezia Arturi, Leonardo Emberti Gialloreti, Mariagrazia Cicala, Francesca Napoli, Cinzia Niolu, Luigi Mazzone, Girolama Alessandra Marfia

**Affiliations:** 1 Department of Biomedicine and Prevention, University of Rome Tor Vergata, Rome, Italy;; 2 Department of Wellbeing of Mental and Neurological, Child Neurology and Psychiatry Unit, Dental and Sensory Organ Health, Policlinico Tor Vergata Hospital, Rome, Italy;; 3 Department of Systems Medicine, Multiple Sclerosis Clinical and Research Unit, Policlinico Tor Vergata, Tor Vergata University, Rome, Italy;; 4 Department of System Medicine, University of Rome Tor Vergata, Rome, Italy;; 5 Department of Wellbeing of Mental and Neurological, Psychiatry and Clinical Psychology Unit, Dental and Sensory Organ Health, Policlinico Tor Vergata Hospital, Rome, Italy

**Keywords:** Multiple sclerosis, pregnancy, children, neurodevelopment, autism, natalizumab

## Abstract

**Introduction:**

Maternal chronic immune and inflammatory conditions may predispose newborns to atypical developmental trajectories, identifying pregnancy as a key period for the etiopathogenesis of neurodevelopmental disorders. The possible long-term impact of maternal multiple sclerosis (MS) on the offspring’s cognitive and behavioral development and its pharmacological treatment during pregnancy is mostly unknown. This study aims to delineate the cognitive and behavioral profile of offsprings exposed to maternal MS, untreated or treated with Natalizumab throughout pregnancy, in comparison to a control group.

**Methods:**

We retrospectively enrolled 39 children (23 males; 16 females; mean age 45.82 ± 35.46 months) exposed to maternal MS, untreated or treated with Natalizumab throughout pregnancy, and 36 children (24 males; 12 females, mean age 38.03 ± 21.52 months) of healthy mothers. All offspring underwent a standardized evaluation of their intellectual or developmental quotient, adaptive functioning, and behavioral issues, including symptoms of autism.

**Results:**

The clinical profile of the included offspring was characterized by an adequate cognitive profile and a good level of adaptive skills (MS offspring: Griffiths III mean total DQ (N = 30) 114.57; WISC-IV mean Full IQs (N= 9) 115.44; mean ABAS GAC 97.28/Control offspring: Griffiths III mean total DQ (N = 31) 105.42; WISC-IV mean Full IQs (N= 4) 119.25 ± 11.32; mean ABAS GAC 97.82 ± 21.4). Furthermore, no significant behavioural problems or autism symptoms emerged in the entire group, regardless of MS treatment.

**Conclusion:**

Offspring's developmental and behavioral phenotypes do not appear to be influenced by maternal treatment with Natalizumab until late pregnancy, nor by maternal variables directly related to MS (age at the time of MS diagnosis, disease duration, and severity).

## 1 INTRODUCTION

The neurodevelopment of a child implies the achievement of motor, language, social, and cognitive milestones and skills. The developmental and behavioral profile of a child is influenced by several factors, genetic and environmental, occurring during the early stages of prenatal and postnatal life.

Atypical developmental profiles can manifest as Neurodevelopmental Disorders (NDDs), defined by the DSM-5 TR [1] as clinical dimensions characterized by early onset, specific cognitive or behavioral symptoms, and social, academic, and work impairment relative to the specific disorder. Some NDDs present clinical manifestations that may also be found in children not affected by such disorders, although with different intensity, frequency, and level of impairment. This highlights the need to investigate NDDs using standardized tools, providing a quantitative and qualitative measure of symptoms.

The etiopathogenesis of NDDs is multifactorial, involving a strong genetic component influenced by several environmental determinants, most of which are still unknown [2-7]. Pre-natal and early post-natal life have been identified as the most vulnerable developmental periods during which the environmental determinants (*i.e*., infections, toxic agents) may contribute to atypical neurobiological development, leading to altered cognitive and behavioral trajectories and, ultimately, to neurodevelopmental disorders (NDDs) in extrauterine life [8-10]. Inflammation and immune response appear to be the primary mechanisms through which the external insult determines a neurodevelopmental vulnerability impacting the newborn developmental trajectory [11-15]. Thus, maternal chronic conditions characterized by inflammation and immune dysregulation (*i.e*., immune disorders, psychiatric and neurological disorders) represent research key topics in the context of NDDs etiopathogenesis [16-24].

Multiple sclerosis (MS) is a chronic autoimmune condition of the central nervous system affecting mostly women in their childbearing age [25-27]. Therefore, women with MS often face considerations regarding pregnancy, occurring in one out of three women after MS diagnosis [28-30]. Accumulating evidence has consistently shown that pregnancy mitigates the risk of relapses [31-33] and that outcomes of infants born to mothers with MS are not significantly different compared to the general population; conversely, little data is available on the long-term outcome of these children, in particular regarding their neurodevelopment [34-38]. In recent years, according to the evolving therapeutic algorithms, more and more women with MS receive disease-modifying treatments (DMTs) at the time of conception. As some treatments, such as Natalizumab, harbour a higher risk of relapse upon discontinuation, continued use during pregnancy up to the third trimester and early resumption after delivery (within 8 to 12 weeks of last dosing) is currently recommended [39-41]. On the subject of fetal development, it has been shown that the use of Natalizumab during pregnancy does not seem to increase the risk of major birth defects, but it can be associated with haematological abnormalities in the newborn, which require monitoring, such as thrombocytopenia and anaemia [42, 43]. Data suggest that while the majority of infants exposed to maternal MS do not show emotional-behavioral problems, the risk of NDDs is yet not widely investigated [34]. Moreover, the impact of disease-modifying therapies (DMTs) taken during pregnancy on the child’s cognitive, emotional, and behavioral development remains unclear.

Upon such background premises, we designed a study aiming to delineate the cognitive and behavioral profile of offspring exposed to maternal MS, untreated or treated with Natalizumab throughout pregnancy. Specifically, the main objectives of the study are:

- To evaluate the offspring’s developmental, cognitive, adaptive, and behavioral profile of a group of women affected by MS in comparison to a control sample of healthy control women and their respective offspring.
- This study aims to evaluate the possible influence of maternal factors, specifically those related to multiple sclerosis, on the developmental and behavioral profiles of their offspring, including illness duration and severity, as well as the use of disease-modifying therapies (DMTs) during pregnancy.

## 2 MATERIALS AND METHODS

### 2.1 Study Design and Enrollment of Participants

This study was conducted as part of a broader project aimed at defining the cognitive, adaptive, and behavioral profiles, as well as the developmental trajectories, of offspring prenatally exposed to maternal chronic conditions. [16].

For the purpose of the present study, we retrospectively enrolled women affected by relaping-remitting MS (WwMS) and their offspring, followed at the MS Pregnancy clinic of the Tor Vergata University Hospital, Rome, Italy, during the period spanning from pregnancy planning to 12 months after delivery.

The control group was constituted of women not affected by MS (and their respective offspring) or other chronic diseases (neurological, psychiatric, and immune function disorders such as diabetes, rheumatoid arthritis, and/or infectious diseases) who were screened for psychiatric disorders during pregnancy as part of the aforementioned project [16]

Women with MS and control participants were retrospectively contacted by phone, at a distance from delivery, to propose the opportunity to perform a screening of their offspring’s developmental and behavioral profile.

Thus, offspring of both groups of mothers (WwMS *vs*. c ontrol) were retrospectively enrolled and clinically evaluated through an in-depth standardized developmental and behavioral assessment by a multidisciplinary team of clinical psychologists and child psychiatrists (see paragraphs below).

All participants (women and legal guardians of offspring included) provided written informed consent. The whole project was approved by the ethical committee (#145/20) of the Policlinico Tor Vergata University Hospital, Rome, Italy.

### 2.2 Mother Evaluation

#### 2.2.1 MS Group

The following clinical information was collected for all the women with MS: date of MS diagnosis, date of last menstrual period (LMP) before pregnancy confirmation and date of delivery; disease duration at delivery; Expanded Disability Status Scale (EDSS) before conception [44]; DMTs before conception, during pregnancy, and after delivery; date of last DMT assumption with respect to last menstrual period (LMP).

Exposure to treatment during pregnancy was calculated as the interval between the last treatment assumption and LMP. Furthermore, the following clinical data regarding pregnancy were gathered: adverse events during pregnancy, type of delivery, adverse events during delivery, and fetal distress. Moreover, we collected APGAR scores that assess the health of newborns at 1 and 5 minutes after birth (normal range 7-10).

#### 2.2.2 Control Group

The control group was clinically evaluated in the context of a previous project [16] through a clinical diagnostic interview focused on demographic and clinical data specifically regarding inclusion/exclusion criteria (*i.e*., neurological, psychiatric, and immune function disorders such as diabetes, rheumatoid arthritis and/or infectious diseases). Control participants were matched with MS women on the basis of age.

Most of the women in the control group (N = 26; 72.2%) showed a high educational level (college), while 8 (22.2%) showed high school education, and just 2 (5.5%) presented a middle school level of education. Only a few women in this group were non-employed (N = 4; 11.11%), while the majority of them had employment (N = 32; 88.89%).

### 2.3 Offspring Evaluation

Offspring belonging to both groups of mothers (MS and Control) underwent a standardized assessment of developmental or cognitive level, adaptive skills, and behavioral profile, with a focus on autism symptoms, through the following standardized tools. Each measure was administered to participants depending on their chronological age (see below).

#### 2.3.1 Developmental, Cognitive, and Adaptive Functioning Evaluation

Offspring aged < 6 years were administered using the third edition of Griffiths Scale [45], which provides a developmental profile of the child, specifically measuring the Developmental Quotient (DQ) in different domains: foundations of learning (scale A), language and communication (scale B), fine (scale C) and gross motor abilities (scale E), personal-social-emotional skills (scale D). The Griffiths III provides specific scores (developmental age, in months, scaled score, development quotient, percentile) in addition to a comprehensive measure of a child’s development (Total DQ). Developmental quotients have a mean of 100 and a standard deviation of 15.

Offspring aged ≥ 6 years underwent an evaluation of the Intellectual Quotient (IQ) through the fourth edition of the Wechsler Intelligence Scale for Children (WISC-IV).

The WISC-IV Scale [46] provides scores in different intellectual domains (including Verbal Comprehension, Perceptual Reasoning, Working Memory, and Processing Speed), as well as a full IQ score (Total IQ). Standard scores are represented by a mean of 100 and a standard deviation of 15.

The evaluation of adaptive skills was performed for all participants using the Adaptive Behavior Assessment System, Second Edition (ABAS-II) [47]. This parent-report questionnaire measures the child’s adaptive skills in several life contexts. Parents are asked to rate their child’s abilities (from 0 = “not able to” to 3 = “able to do it and always performs it when needed”) in 10 different adaptive domains (*i.e*., communication, use of the environment, preschool competences, domestic behavior, health and safety, play, self-care, self-control, social abilities, and motility). Scores of each domain are summarized in four adaptive composite scores, which present a mean of 100 and a standard deviation of 15: Conceptual Adaptive Domain (CAD), Practical Adaptive Domain (PAD), Social Adaptive Domain (SAD), and General Adaptive Domain (GAC). The test is composed of two age forms, chosen based on the participant’s chronological age: “0-5 years” or “5-21 years” form.

#### 2.3.2 Behavioral Evaluation

The behavioral and emotional profiles of all offspring included in the study were assessed using the parent-report Achenbach Child Behavior Checklist (CBCL) [48]. The CBCL includes two different forms, depending on the child’s age: the “18 months-5 years” form or the “6-18 years” form. Parents are asked to rate their child’s behavior on a three-point Likert Scale (0 = not true, 1 = sometimes true, 2 = often true), considering the frequency of the behavior. Lower scores indicate less problematic behavior, while higher scores identify difficulties in behavior or emotional profiles. The behavior is classified as typical (T score < 65), borderline (T = 65-69), or clinically significant (T ≥ 70). The two age forms of the CBCL include several items organized in different scales; however, both forms summarize these scales in three main domains: internalizing, externalizing, and total problems. For this study, we considered only these three domains.

#### 2.3.3 Autism Symptoms Assessment

In order to investigate the presence of autism symptoms, the second edition of the Autism Diagnostic Observation Schedule (ADOS-2) was performed in accordance with the criteria of the Diagnostic and Statistical Manual of Mental Disorders, Fifth Edition (DSM-5-TR) [1].

The ADOS-2 [49] is the gold standard tool for the assessment of autism symptoms. It is organized into different modules, depending on the child’s age and expressive language level: Toddler Module (for children aged between 12 and 30 months, able to walk independently for five steps), Module 1 (for children aged ≥ 31 months, with preverbal abilities), Module 2 (for children with non-fluent language abilities, although including sentences), Module 3 (for children and adolescents up to 16 years of age, with fluent language), Module 4 (for adolescents aged > 16 years and adults). For the purpose of this study, we used only Toddler Modules 1, 2, and 3.

The ADOS-2 provides the following scores: Social Affect (SA), Restricted and Repetitive Behavior (RRB), and Total (AS + RRB). In modules 1 to 3, the total score is converted into a Calibrated Severity Score (CSS), which gives information regarding the level of autism symptoms severity (ranging from 1 to 10, with higher scores indicating more severe autistic symptoms). In the Toddler Module, the total score is converted into a risk category. Additionally, it is possible to obtain the CSS using a specific conversion table [50].

### 2.4 Statistical Analysis

The statistical analysis included a comprehensive descriptive examination of the entire sample of women and their offspring. For categorical variables, the number of observations and the proportion for each category were calculated. Continuous variables were summarized using mean values and standard deviation (SD).

The calculation of the offspring sample size, aimed at detecting a difference of at least 10 points in DQ, IQ, or developmental and adaptive function (ABAS), was performed considering a power of 0.8 and an alpha error of 0.05.

Comparison between WwMS and controls (regarding age at delivery and age at child’s assessment) were analyzed using independent samples t-test.

The Mann-Whitney nonparametric test was employed to evaluate differences between the two groups of offspring in terms of age at assessment, DQ, IQ, adaptive scores, developmental and behavioral profile, including autism symptoms. An alpha level of 0.002 was used after Bonferroni correction for multiple comparisons.

Bivariate Spearman’s correlations were applied to estimate the relationship between maternal factors related to maternal MS (*i.e*., age at MS diagnosis, disease duration, disability before first pregnancy) and the offspring’s developmental and behavioral profile.

Comparison between MS offspring exposed to natalizumab during pregnancy and those not exposed were analyzed using the Mann-Whitney nonparametric test, focusing on DQ_IQ, adaptive scores, autism symptoms, behavioral profile, and auxological parameters at birth. Unless otherwise specified, an alpha level of 0.05 was used for the statistical analysis.

All statistical analyses were performed using SPSS v.26.0 (IBM Corp.).

## 3 RESULTS

### 3.1 Maternal Demographic and Clinical Data

The final sample of the study was constituted of 68 women (32 WwMS giving births to 39 infants; 36 controls), with a mean age of 36.37 ± 4.86 years.

Specifically, from a total of 70 WwMS who were invited to take part in the study, 21 declined, 12 did not respond, and 5 accepted but, due to personal problems, could not show up on the day of the visit.

Therefore, we included 32 WwMS who were afterward matched with a control group composed of 36 women without MS (35.81 ± 5.57 years).

WwMS group showed a mean age of 27.34 ± 4.2 years at disease diagnosis and low disability before conception (EDSS M 1.09 ± SD 0.69; median 1, IQR 0.5).

The two groups, WwMS and controls, did not differ significantly in terms of age at delivery (MS 33.16 ± 4.3 vs. Control 32.86 ± 5.4 years *p* = 0.808) and age at child’s assessment (MS 36.38 ± 4.8 vs Control 35.81 ± 5.5 *p* = 0.657).

#### 3.1.1 MS Pregnancies

As previously described, we have finally included 32 women with MS (WwMS). However, 6 of them had more than one pregnancy, thus more than one child. For these reasons, we provide information about MS pregnancies (N = 39) instead of MS women (N = 32) (Table **1**), with reference to the following time periods: before conception and during each trimester of gestation.

#### 3.1.2 Exposure to DMTs before Conception and During Pregnancy

All the WwMS enrolled were under treatment before pregnancy and at the time of conception (Table **1**), with different medications, including Natalizumab, interferon beta, glatiramer acetate, dimethyl fumarate, fingolimod, ocrelizumab.

Among these, the majority discontinued the other DMTs upon confirmation of pregnancy, and the others were treated with Natalizumab during pregnancy.

In pregnancies exposed to NTZ, the median time of exposure was 200 days (min 97-max 207), with the last infusion administered at the median gestational week of 28.5 In the group of women treated with other DMTs, median exposure was 32 days (min -237-max 140), including both women who received the last dose of treatment before LMP and after LMP.

None of the women in the group not exposed to NTZ experienced relapses during pregnancy, except for one.

### 3.2 Offspring Demographic Data

A sample size of at least 35 offspring per group was calculated.

The offspring total sample was constituted of 75 children, with a mean age at evaluation of 42.08 months ± 29.6 (median age 36 months): 39 children (23 males; 16 females, mean age of 45.82 ± 35.46 months (median age 40 months) born to mothers with MS (MS_offspring) including one pair of twins, four pairs of siblings and one case of three brothers; 36 children (24 males; 12 females; mean age of 38.03 ± 21.52 months (median age 36 months)) born to control mothers (Control_offspring). The two groups of offspring did not significantly differ in terms of age at the assessment (*p* = 0.477).

#### 3.2.1 MS_offspring

Concerning clinical data, an eutocic delivery was reported in 23 (59%) newborns, while adverse events during delivery were described in 8 (20.5%) and fetal distress in 7 newborns (17.9%). Moreover, APGAR scores were 9 or 10 in 20 (87%) at the first minute and in 21 at the fifth minute (91.3%). APGAR scores below the score of 9 range were found in 3 babies (13%) at the first minute and in 2 babies (8.7%) at the fifth minute.

### 3.3 Results Objective A: Neurodevelopmental Assessment in MS and Control Offspring

The developmental quotients (DQs) of MS offspring, as measured by the Griffiths III (N = 30), showed normal scores in all the domains (scale A foundations of learning 114.20 ± 11.94; scale B language and communication 100.57 ± 12.15; scale C fine motor abilities 113.4 ± 13.19; scale D personal-social-emotional skills 115.77 ± 14.55; scale E gross motor abilities 121.33 ± 15.95; global developmental quotient 114.57 ± 12.02). Full IQs, evaluated through the WISC-IV (N= 9), revealed scores in the normal threshold (115.44 ± 23.45). Parents reported all adaptive skills domain in line with their offspring’s chronological age: General (GAC 97.28 ± 13.72), Conceptual (CAD 99.95 ± 14.90), Social (SAD 99.23 ± 15.09), Practical (PAD 92.85 ± 14.98).

Regarding the behavioral profile, we found a low level of autism symptoms as measured by the CSS of the ADOS-2 (2.06 ± 1.53) and a lack of behavioral difficulties in all the domains investigated (CBCL internalizing 47.39 ± 11.70; CBCL externalizing 47 ± 10.26: CBCL total 45 ± 11.75).

The developmental quotients (DQs) of control offspring, as measured by the Griffiths III (N = 31), resulted in normal scores in all the domains (scale A 107.84 ± 19.94; scale B 100.23 ± 19.29; scale C 102.32 ± 19.25; scale D skills 105.61 ± 20.43; scale E 103.97 ± 25.71; Total DQ 105.42 ± 19.91).

Full IQs, evaluated through the WISC-IV (N= 4), revealed scores in the normal threshold (119.25 ± 11.32). Parents reported all adaptive skills domains in line with their offspring chronological age: GAC 97.82 ± 21.4; CAD 104.24 ± 18.71; SAD 100.47 ± 19.57; PAD 94.35 ± 24.38.

The behavioral evaluation, including autism symptoms, did not reveal any significant difficulties (ADOS-2 CSS 2 ± 1.74; CBCL internalizing 49.59 ± 11.17; CBCL externalizing 46.81 ± 11.10: CBCL total 41.11 ± 11.16).

#### 3.3.1 Results Objective A: MS Offspring vs. Control Group

Concerning developmental, cognitive, and adaptive skills, we did not find any statistically significant differences between the two groups in terms of DQ, IQ, and adaptive scores (Table **2**). The level of autism symptoms (ADOS-2 CSS) did not differ among the off spring of mothers affected or not by MS.

Likewise, behavioral evaluation showed no statistically significant differences between the two groups in the Internalizing score, neither in the Externalizing score nor in the Total Problems score (Table **2**).

### 3.4 Results Objective B

Concerning objective B, given that the statistical unit of the analysis was women with MS, we considered the first pregnancy of each WwMS. Thus, the results reported below regard 32 first pregnancies of 32 MS women (mean age of 36.37 ± 4.86 years at the moment of child’s evaluation; mean age at delivery 33.15 ± 4.37; mean age of 27.34 ± 4.26 years at disease diagnosis) and their respective 32 offspring (14 F, 18 M, mean age 44.31 ± 37.09 months).

#### 3.4.1 Correlations between Maternal Factors Strictly Related to Maternal MS Condition and the Offspring’s Developmental and Behavioral Profile

We analyzed the following MS-related variables: age at MS diagnosis, disease duration (from diagnosis until delivery), and EDSS score in correlation with offspring clinical phenotype (cognitive/developmental, adaptive, and behavioral profiles), summarized in Table **3**.

Specifically, we found no statistically significant correlation between the offspring’s DQ or IQ and age at MS diagnosis, disease duration, and EDSS score before conception. Moreover, offspring adaptive skills (ABAS_GAC) were not related to age at MS diagnosis, disease duration, or EDSS score before conception.

Furthermore, no correlation emerged between maternal factors and offspring's behavioral profile in terms of autistic symptomatology (ADOS_CSS) and in terms of emotional and behavioral difficulties (CBCL scores), neither regarding the age of MS diagnosis nor illness duration or illness severity before conception.

#### 3.4.2 Offspring Exposed versus not Exposed to Natalizumab During Pregnancy

Regarding the MS pharmacotherapy administration during pregnancy, we evaluated the possible impact of in-utero Natalizumab exposure on the cognitive and behavioral profile of the offspring.

First of all, we compared the group of offspring born from mothers who received Natalizumab pharmacotherapy during pregnancy (N = 9) and the group not exposed to the use of Natalizumab (N = 23) without finding any statistically significant differences in terms of DQ/IQ (*p* = 0.557), adaptive scores (*p* = 0.085), autism symptoms (*p* = 0.543) and behavioral profile (Internalizing *p* = 0.670; Externalizing *p* = 0.555; Total *p* = 0.229) (Table **4**). Specifically considering auxological parameters at birth, we found no differences between the two groups: APGAR 1’ (*p* = 0.428), APGAR 5’ (*p* = 0.632), head circumference (*p* = 0.359), weight (*p* = 0.147), and length (*p* = 0.154).

## 4 DISCUSSION

The primary objective of this study was to investigate, using a standardized approach, the developmental and behavioral profiles of a group of offspring born to women affected by MS compared to healthy mothers in order to explore whether or not maternal MS predisposes to developmental and behavioral dysfunction.

The main result of the study is that the developmental and behavioral profile of the included MS offspring was characterized by an adequate developmental or intellectual quotient (according to the age of the participant) in association with a normal level of adaptive skills (child's skills level of independence) and the absence of significant behavioral problems (internalizing such as anxiety, isolation; externalizing such as hyperactivity, impulsiveness, aggressiveness) and autism symptoms.

This clinical phenotype did not significantly differ from the control group for all the clinical domains investigated (developmental quotient, intellectual quotient, adaptive skills, behavioral profile including autism symptoms, externalizing and internalizing behaviors). In addition, none of the maternal MS clinical factors (age at MS diagnosis, disease duration, or disability) had a statistically significant correlation with the behavioral, adaptive, and developmental profile of their offspring. Our results align with previous observations in an independent cohort, showing that maternal MS is not a risk factor for neurodevelopmental disorders in offspring [35, 36, 51-53]. However, our research is hardly comparable with these studies, which were based on retrospective data collection (health registries and databases) without the employment of standardized measures of child development and behavior [35, 37, 51, 52]. Moreover, in the existing literature on this topic, the main child outcomes have primarily focused on vulnerability factors, developmental milestones, anthropometric parameters, or pediatric developmental abnormalities.

Whereas, within our case-control study, we provide an offspring’s detailed developmental and behavioral profile, revealed by standardized direct clinical evaluation and parental report. In addition, we provide a snapshot of the cognitive and behavioral outcomes of offspring at different stages rather than focusing solely on the neonatal period, as done in most previous studies [54-56]. This is essential as it is known that different cognitive, motor, linguistic, communicative, relational, behavioral, and emotional skills are achieved at different stages of development, particularly in neurodevelopmental disorders screening [57-62]. The absence of a developmental and behavioral dysfunction in offspring exposed to the maternal condition of MS represents a main finding with life implications for women affected by a chronic autoimmune condition such as MS. These results may guide neurologists in the counseling for women with MS who are planning a pregnancy, contributing to reduce the stigma of the disease.

Moreover, traditionally, one of the main concerns regarding having a pregnancy in women with MS is the fear of the negative impact of DMTs on offspring. Accumulating evidence supporting the safety of DMTs exposure during pregnancy regarding perinatal outcomes led to a sharp increase in exposure during pregnancy to DMTs over the last decade, However, the safety profile on long-term outcomes has not been fully established. Conflicting opinions, in fact, exist regarding the impact of DMTs use during pregnancy and offspring’s NDD, particularly Natalizumab. In fact, three cases (one singlet and one 5-year-old dizygotic twin couple) [51, 63, 64] of ASD and one of speech disorders [63] were described among offspring exposed to NTZ during early gestation. Moreover, moderate behavioral disturbances were reported as the most frequent developmental anomalies among offspring born from mothers treated with NTZ before or during early pregnancy [65]. Our results, instead, suggest, at a preliminary level, that prolonged antenatal exposure (last administration around the 29^th^ gestational week) to NTZ has no impact on the offspring’s neurodevelopment and/or behaviors. Although larger studies are needed to draw definitive conclusions, such preliminary findings, in addition to data previously published [42, 65-71] reassure fears of increased risk of developmental anomalies due to prolonged exposure to treatment during pregnancy [72].

Such concerns were based on the fact that NTZ blocks the binding of α4 integrins to their ligand VCAM-1, which are important not only for the immune system functioning but also play a role during embryogenesis; in fact, they are expressed in several embryonic tissues including neural crest. On the other hand, interruption of NTZ before conception or during early gestation leads to timely reactivation of peripheral and central inflammation during the initial pregnancy stages [63, 68, 73] when most of the embryogenesis occurs. However, it is known that maternal immune activation (MIA) during pregnancy is associated with an increased risk for neurodevelopmental disorders (NDDs), probably due to the interference of inflammatory cytokines with the neuronal growth of the fetus [74, 75]. Several studies instead consistently showed that continuation of NTZ during pregnancy protects mothers from disease rebound [39, 70]; therefore, we can speculate that continuous exposure to NTZ by controlling excessive maternal inflammation might also indirectly contribute - rather than be deleterious - to normal neurodevelopment of the fetus in women with autoimmune conditions. Indeed, none of the women included in the NTZ group had relapses during pregnancy.

Larger studies are needed to explore this hypothesis, but this is the first work specifically addressing the question of the safety of long exposure to NTZ with respect to offspring’s neurodevelopmental and behavioral profiles.

It is worth highlighting that in our cohort, we did not observe a higher risk of NDD also in women interrupting treatment at early pregnancy (median of exposure 4 weeks). Both for platform therapies and especially for other highly effective treatments (*i.e*., ocrelizumab), the immunological efficacy is expected to persist for several weeks after the last drug administration, supporting the idea of carryover immunomodulation possibly useful to mitigate MIA, in addition to the physiological hormonal driven down-regulation of the immune system physiologically occurring during pregnancy [76]. Interestingly, in this group, only one child was affected by autism spectrum disorder, and this child was born to a woman with MS who experienced a relapse during the first month of gestation. However, the correlation between these two events is by far speculative and deserves confirmation by future studies.

## LIMITATIONS

As a whole, our results are certainly influenced by the small sample size and by the retrospective enrollment of MS women, leading to the selection of women treated with drugs currently less administered.

Specifically concerning the use of DMTs during pregnancy, the main limit of the study is represented by the small sample size of women treated with DMT, which can hardly lead to conclusions.

However, considering the lack of studies employing a good and standardized methodology for child evaluation, we strongly believe that our work, even if at a preliminary level, represents a key contribution, with important implications, to the definition of the offspring’s developmental and behavioral profile of women affected by MS.

## CONCLUSION

Pregnancy and motherhood present significant challenges for all women, even in the absence of chronic conditions, due to concerns about the newborn’s development and well-being. Therefore, offering women with MS evidence-based information about their offspring’s potential developmental outcomes, particularly in relation to the use of DMTs during pregnancy, is undoubtedly a crucial and sensitive issue.

Within this work, we provide preliminary but promising results on the clinical developmental, cognitive, adaptive, and behavioral profile of offspring exposed to maternal MS during pregnancy.

The main finding of this study is the confirmation of adequate cognitive and adaptive skills, along with the absence of significant internalizing or externalizing behavioral problems and autism symptoms.

In addition, even if at a very preliminary level, our data show that, within our sample, there is no increased risk of developmental or behavioral difficulties in mothers with MS who were pharmacologically treated with NTZ until late pregnancy. Noteworthy is the finding that offspring's development and behavioral phenotypes do not seem to be influenced by variables strictly related to maternal MS (age at the time of MS diagnosis, disease duration, and severity).

Taken together, all these results have important implications and an immediate impact on MS women who are planning a pregnancy. As a matter of fact, even if these data need to be replicated, they can support the clinician during the counseling.

However, future research on larger samples is necessary to confirm our data and to provide women with information regarding the possible outcomes of their offspring's development profile, especially regarding the use of DMTs during pregnancy.

## Figures and Tables

**Table 1:** Main characteristics of MS pregnancies (N=39) and control group’s age at delivery.

-	**Total MS** **N = 39**	**Not Exposed to NTZ** **N = 28**	**Exposed to NTZ** **N = 11**	**Control Group** **N = 36**
**Age at delivery (yrs) (M ± SD)**	33.07 ± 4.30	33.35 ± 4.45	32.36 ± 4.01	32.86 ± 5.47
**Disease duration until delivery (yrs) (M ± SD)**	6.31 ± 3.32	6.14 ± 3.53	6.73 ± 2.83	-
**EDSS (M ± SD)**	1.12 ± 0.68	1.20 ± 0.67	0.91 ± 0.70	-
**DMTs at conception** IFN Glatiramer Acetate Dimethyl Fumarate Ocrelizumab Fingolimod Natalizumab	30.8% (n=14) 20.5% (n=8) 7.7% (n=3) 7.7% (n=3) 5.1% (n=2) 23.1% (n=9)	42.9% 28.6% 10.7% 10.7% 7.1% 0%	- - - - - 100%	-

**Abbreviations**: EDSS: Expanded Disability Status Scale; DMTs: disease-modifying treatments; IFN: Interferon beta; NTZ: Natalizumab.

**Table 2:** Results of the offspring evaluation in both groups (MS offspring and control group).

**-**	**MS_offspring** **(N=39)** **Me (IQR)**	**Control Group_offspring** **(N=36)** **Me (IQR)**	***U di Mann-Whitney***	***p***
Age (in months)	40 (46)	36 (44.25)	769.000	0.477
DQ_scale A	117 (14.0)	108 (19.5)	570.500	0.128
DQ_scale B	102 (17.3)	102 (25.5)	422.500	0.539
DQ_scale C	114 (19.8)	106 (23.0)	629.000	0.018
DQ_scale D	119 (16.5)	105 (28.0)	592.000	0.067
DQ_scale E	123 (24.5)	103 (29.5)	666.500	**0.004**
Global DQ	117 (14.8)	111 (17.0)	606.500	0.041
IQ_TOT	117 (22.0)	119 (15.3)	16.500	0.825
DQ/IQ	117 (17.5)	112 (17.0)	805.000	0.068
ABAS_GAC	98 (13.0)	94.5 (26.8)	708.000	0.618
ABAS_CAD	101 (13.5)	102 (19.5)	632.500	0.736
ABAS_SAD	96 (19.0)	101 (29.0)	643.500	0.829
ABAS_PAD	94 (17.0)	87.0 (25.5)	713.500	0.576
ADOS-2 CSS	2 (1.0)	1 (1.75)	596.500	0.424
CBCL_INT	48 (13.3)	49 (16.5)	447.000	0.587
CBCL_EXT	47.5 (17.0)	46 (15.0)	497.500	0.873
CBCL_TOT	42.5 (14.5)	37 (8.0)	598.500	0.118

**Abbreviations**: Me(IQR): median(interquartile range); DQ: Developmental Quotient measured by Griffiths III, scale A: foundations of learning, scale B: language and communication; scale C: fine motor abilities, scale D: personal-social-emotional skills, scale E: gross motor abilities. IQ: Intellectual Quotient measured by the Wechsler Intelligence Scale for Children. Adaptive functioning is measured with the Adaptive Behavior Assessment System, Second Edition (ABAS-II): General (GAC), Conceptual (CAD), Social (SAD), and Practical (PAD). Autism symptoms measured by ADOS-2 CSS: Autism Diagnostic Observation Schedule Calibrated Severity Score. Behavioral evaluation performed by: Child Behavior Checklist Internalizing score (CBCL_INT), Externalizing score (CBCL_EXT) and Total Problems score (CBCL_TOT).

**Table 3:** Bivariate Spearman’s correlation analysis between maternal factors related to MS and the offspring’s developmental and behavioral profile.

**-**	**-**	**Age at MS Diagnosis**	**Illness Duration**	**EDSS Score Before Conception**
DQ/IQ	Rho *p*-value	0.141 0.443	-0.088 0.631	-0.154 0.399
ABAS_GAC	Rho *p*-value	-0.127 0.490	0.025 0.894	-0.169 0.356
ADOS-2 CSS	Rho *p-*value	-0.125 0.517	0.013 0.947	0.210 0.275
CBCL_INT	Rho *p*-value	-0.017 0.929	-0.245 0.201	-0.051 0.795
CBCL_EXT	Rho *p-*value	-0.006 0.974	0.008 0.968	-0.026 0.895
CBCL_TOT	Rho *p*-value	-0.143 0.459	-0.003 0.989	-0.133 0.492

**Abbreviations**: DQ: Developmental Quotient; IQ: Intellectual Quotient; ABAS_GAC: General Adaptive Composite; ADOS-2 CSS: Autism Diagnostic Observation Schedule Calibrated Severity Score; CBCL_INT: Child Behavior Checklist Internalizing score; CBCL_EXT: Externalizing score; CBCL_TOT: Total Problems score.

**Table 4:** Comparison between offspring of MS women treated or not treated with Natalizumab during pregnancy.

**-**	**Not Exposed to Natalizumab** **(N = 23)**	**Exposed to Natalizumab** **(N = 9)**	***U di Mann-Whitney***	***p***
**DQ/IQ** (M ± SD)	113.87 ± 17.59	117.22 ± 8.14	89.0	0.557
**ABAS_GAC** (M ± SD)	94.48 ± 13.84	105 ± 14.40	62.0	0.085
**ADOS-2 CSS** (M ± SD)	2.15 ± 1.84	2.11 ± 1.27	77.5	0.543
**CBCL_INT** (M ± SD)	46.90 ± 12.13	48.22 ± 13.21	80.5	0.670
**CBCL_EXT** (M ± SD)	45.55 ± 10.67	47.67 ± 10.49	77.0	0.555
**CBCL_TOT** (M ± SD)	43.15 ± 11.49	48.56 ± 12.15	64.0	0.229

**Abbreviations**: DQ: Developmental Quotien; IQ: Intellectual Quotient; ABAS_GAC: General Adaptive Composite; ADOS-2 CSS: Autism Diagnostic Observation Schedule Calibrated Severity Score; CBCL_INT: Child Behavior Checklist Internalizing score; CBCL_EXT: Externalizing score; CBCL_TOT: Total Problems score.

## Data Availability

All the data and supporting information is provided within the article.

## LIST OF ABBREVIATIONS

CAD: Conceptual Adaptive Domain
CSS: Calibrated Severity Score
DMTs: Disease-modifying Treatments
GAC: General Adaptive Composite Domain
LMP: Last Menstrual Period
MIA: Maternal Immune Activation
NDDs: Neurodevelopmental Disorders
PAD: Practical Adaptive Domain
RRB: Restricted and Repetitive Behavior
SA: Social Affect
SAD: Social Adaptive Domain
